# COVID-19 and Pregnancy

**DOI:** 10.15190/d.2022.6

**Published:** 2022-06-30

**Authors:** Selia Chowdhury, Mehedi Hasan Bappy, Shreeya Desai, Samia Chowdhury, Vraj Patel, Md. Shahraj Chowdhury, Ayesha Fonseca, Catalina Sekzer, Samina Zahid, Athanasios Patousis, Anna Gerothanasi, Matias Juan Masenga

**Affiliations:** ^1^Dhaka Medical College, Dhaka, Bangladesh; ^2^University of Iowa, Iowa City, IA, USA; ^3^Pramukhswami Medical College, Karamsad, India; ^4^Sylhet MAG Osmani Medical College, Sylhet, Bangladesh; ^5^Smt. NHL Municipal Medical College, Ahmedabad, India; ^6^DSHE, Education Ministry, Sylhet, Bangladesh; ^7^AUIS School of Medicine, Barbados; ^8^University of Buenos Aires, Argentina; ^9^Khyber Girls Medical College, Peshawar, Pakistan; ^10^Georgios Papanikolaou General Hospital, Thessaloniki, Greece; ^11^Aristotle University of Thessaloniki, Thessaloniki, Greece

**Keywords:** COVID-19, pregnancy, newborns, SARS-CoV-2, management, vaccination, labor guidelines.

## Abstract

It is of greatest concern how COVID-19 is affecting pregnancy, mothers, and babies. Scientists are studying the impact of COVID-19 on pregnant women and babies and are understanding a little more every day. Reports show that there is an increased risk in pregnant women compared to nonpregnant women to get more serious illness due to COVID-19. Researchers are also investigating COVID-19 and its potential impact on a fetus. There are exceedingly rare cases of COVID-19 transmission to the fetus, and newborns can pick up COVID-19 when exposed. Vaccines are proved to be safe for pregnant women and help prevent both mother and the fetus from getting COVID-19 and are also highly effective to prevent COVID-19 infection, critical sickness, and fatalities in general. There are specific guidelines for labor and delivery during the COVID-19 pandemic which are to be imposed and followed to achieve safer and healthier childbirth. In this article, the overall influence of COVID-19 in pregnancy, its pathophysiology, effects on placenta and neonates, maternal and perinatal features and outcomes, the role of vaccination, available treatment options, and the guidelines to be followed during the pandemic are discussed based on the available scientific evidence.

## SUMMARY

*1. Introduction*

*2. Pathophysiology*

*3. Effect on placenta*

*4. Vertical transmission*

*5. Fetal and neonatal impact*

*6. Perinatal outcomes*

*7. Role of vaccination*

*8. Currently available treatment options, their efficacy and safety*

- *8.1. Antivirals*

- *8.2. Immunomodulatory agents*

- *8.3. Neutralizing antibodies*

- *8.4. Thromboprophylaxis*

*9. Labor and delivery guidance*

*10. Conclusion*

## 1. Introduction

SARS-CoV-2 is a novel coronavirus and the cause of the ongoing COVID-19 pandemic. The transmission is mainly through aerosols, respiratory droplets, and fomites. Commonly, it causes fever, dry cough, shortness of breath, headache, and fatigue^1,2^. More specific symptoms are also reported, such as anosmia and dysgeusia. Although it usually has a benign course, potential complications including severe pneumonia, respiratory failure, hypercoagulability, shock, and organ failure are also seen^3^. This novel virus enters the host cell by attaching its viral spike protein to the human receptor angiotensin-converting enzyme 2 (ACE2)^4^. This enzyme is essential for the correct regulation of the physiological renin-angiotensin-aldosterone system (RAAS), and it is expressed in several organs, such as the kidney, lung, heart, and placenta^5^. Although ACE2 is essential for SARS-CoV-2 entry into human cells, it is shown that it has some protective effects on the severity of the disease, mediating mainly against acute severe lung injury and balancing pro-inflammatory and anti-inflammatory function of RAAS. Data shows that ACE2 levels vary between people. Young people and women have higher levels of ACE2 whereas having risk factors, such as diabetes decreases these levels. Genetic phenotype is important as well. For example, East-Asian females have significantly higher expression of ACE2. This may somehow explain why younger people have a milder presentation of the infection, as well as why elderly men with risk factors have the worst outcome^5^. Pregnant women are at higher risk of developing severe symptoms when infected with SARS-CoV-2, with a greater percentage of pregnant women needing ICU admission. The evidence seems to show an extensive immune reaction in the placenta, such as villous hyperplasia, and mural hyperplasia. This is observed as chorioangiosis, fetal thrombosis, chorioamnionitis, and chronic villitis and represents an increased risk of undesirable outcomes during pregnancy and labor such as spontaneous preterm birth and higher maternal mobility^6^. Finally, the World Health Organization (WHO), the Centers for Disease Control and Prevention (CDC), and the American Academy of Pediatrics (AAP) recommend breastfeeding while minimizing the risk of transmission such as a good hand wash before contact, and the use of a face mask, will ensure correct nutrition for the baby. Furthermore, studies confirmed that SARS-CoV-2 antibodies are transmitted through breastmilk, while the virus was undetected^6^.

## 2. Pathophysiology

**Figure 1:**
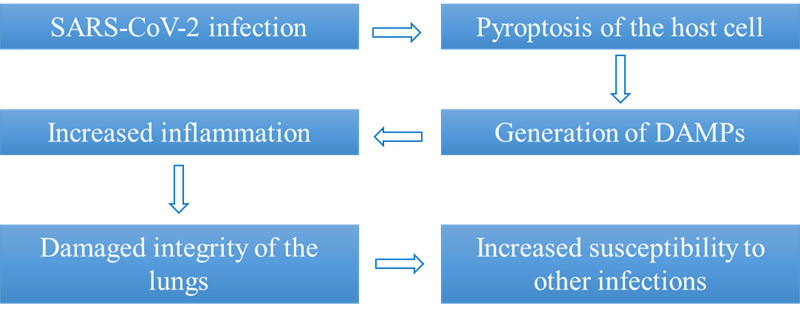
Mechanism of increased inflammation in SARS-CoV-2 infection

The response to infections, particularly viruses, may be affected by changes in the maternal immune system during pregnancy. It is considered that, at least in part, the enhanced inflammatory response to viruses during pregnancy is mediated by the following factors: (1) There is a change in the CD4+ T cell population toward the Th2 phenotype rather than the Th1 phenotype. (2) There is a decrease in the number of circulating natural killer (NK) cells. However, whether this decrease in circulating NK cells has clinical ramifications for COVID-19 remains unknown. (3) There is a reduction in plasmacytoid dendritic cells (pDCs) in the circulation. These cells are essential for the generation of type 1 interferon, which protects against viruses. Furthermore, it has been discovered that pDCs from pregnant women have a reduced inflammatory response to the H1N1/09 virus. (4) The innate immune system undergoes changes, including the pattern recognition receptors Toll-like receptors (TLRs)^7^. SARS-CoV-2 infection produces host cell pyroptosis (programmed cell death triggered by inflammation in response to a pathogenic stimulus) and the generation of damage-associated molecular patterns (DAMPs), which can act as TLR ligands and increase inflammation (Figure 1). These changes in the maternal immune system have ramifications for COVID-19's clinical course, as well as its treatment and prevention during pregnancy. However, whether these modifications increase susceptibility and/or morbidity or are protective against COVID-19 must be discovered^7^. In most cases, the placenta acts as a barrier to prevent maternal infection from passing to the fetus (vertical transmission). SARS-CoV-2 expression has been found in midtrimester placenta samples; however, it is unclear if the virus was there owing to primary infection or was aided by placental damage caused by other illnesses. The mechanisms of viral infection of the placenta are still unknown. Given the lack of coexpression of ACE2 and transmembrane serine protease 2 (TMPRSS2) in the placenta, it appears likely that SARS-CoV-2 penetrates the placental tissues through a different route. Other proteases have also been linked to the problem. Both DPP4 and CD147 are extensively expressed in the placenta during pregnancy, suggesting that they may play a role in cell entrance. Although neonatal positivity at birth was inconsistent, SARS-CoV-2 viral RNA was found in the amniotic fluid in case reports of significant maternal sickness. Future research should investigate the inflammatory response, viral load, antibody production, and level of immunity developed in pregnant women at various gestational ages^7^.

## 3. Effect on placenta

The COVID-19 pandemic has had an extreme impact on the population’s health. There are many ongoing investigations and recent studies every day regarding how the SARS-CoV-2 virus behaves in the general population. Studies that analyze pregnant patients and newborns must consider several different variables. Not all mothers are the same, and neither are their pregnancies. Therefore, this virus still presents a challenge to all doctors and researchers. COVID-19 infections in pregnant women have been shown to increase the risk of adverse outcomes during pregnancy such as alterations in perfusion^8^, vasoconstriction, intrauterine growth restriction (IUGR), and eventually preterm birth^9^. Many of the infected women had to go through a preterm emergency cesarean section because of fetal distress^8,9^. Studies were conducted on mothers, during pregnancy, and after labor. Most of the trials investigated how the virus could infect the placenta cells and how this would affect the ongoing pregnancy and if this could have an impact on the health of the mother and newborn. Many studies began by taking samples of the placental tissue and focusing on the interaction between the virus and the cellular receptor. Like lung tissue, the placenta also owns ACE2 receptors^8,10^. This receptor plays a key role in the entry and replication inside lung tissue and placental tissue as well^11^. This could be thought to be one of the most important histopathological findings^8,10,11^. This receptor is also a component of the ACE-AngII-AT1 axis^8^. There is a theory that if the virus interferes with this placental receptor, it may contribute to the pathological findings of vasoconstriction, fibrosis, inflammation, and thrombo-embolic processes in the placenta^8^. Once pregnant women have been infected with the virus, it can generate a diffuse and severe inflammatory response in the placenta. This ongoing inflammation can lead to what has been shown to be a local inflammatory response, an increase in fibrin deposition, mediated by B-cells, T-cells, and macrophages^12,13^. The main target of the virus is considered the syncytiotrophoblast cells that are a component of the placenta^14^. These histopathological findings could be compared to the changes found in the lung tissue of patients who are not pregnant and could be considered the counterpart of the diffuse alveolar damage found in patients with pneumonia by SARS-CoV-2^11-14^. Other hypotheses propose that a different receptor could play a role in the susceptibility of placental cells to SARS-CoV-2 infection. This receptor, named “caveolin” is thought to endocyte certain viruses^15^. Since syncytiotrophoblasts lack this membrane structure, there would not be an inflammatory response that would result in the syncytiotrophoblast remaining intact, with no alteration of its structure and thus SARS-CoV-2 cannot cross into the placental villi^15^. In addition, there are many hypotheses that these placental changes could be a result of maternal hypoxemia since the mother has an ongoing infection^15^. The SARS-CoV-2 virus has been shown to generate systemic inflammation that could alternate the perfusion and as a result the function of the placental cells. If the placenta is severely damaged, it can lead to diffuse inflammation, chronic intervillositis, and necrosis of the cells^15^. In brief, the infection of SARS-CoV-2 during pregnancy can lead to a variety of microscopic changes in the placenta. These changes could interfere with local perfusion, the most important function of the placenta. This would increase the risk of IUGR and eventually preterm birth. Results are still very inconclusive, and there is much more to learn about the effects at the placental level.

## 4. Vertical transmission

After nearly two years of the ongoing pandemic, research studies have been conducted all over the world regarding the SARS-CoV-2 virus. The transmission of this highly contagious virus via respiratory droplets has been a topic of great discussion regarding vertical transmission from pregnant women infected with SARS-CoV-2 and newborns. Nonetheless, transmission rates from mother to fetus during pregnancy in various studies have shown to be low, transmission rates are thought to be between 0.5% and 2.5%^16^. Studies were conducted in order to determine transmission between the mother and the fetus. It is well known that an infected mother can transmit the virus through respiratory droplets during breastfeeding if the appropriate protection measures are not taken. If the correct measures are taken, such as wearing surgical masks when breastfeeding, then breastfeeding and vaginal delivery are unlikely to transmit the virus^17^. The transplacental theory is the theory that stands until today over the others. Studies were conducted to determine transplacental infection. In various studies, samples were taken from the mothers and fetal blood and from the placenta. In a single case study conducted in Renmin Hospital blood samples drawn from the baby after delivery showed elevated IgM antibody levels but negative RT-PCR from nasopharyngeal swabs from the newborn^18^. The elevated IgM level, that must be produced by the fetus and cannot be passed on from the mother to the neonate, could suggest that the infection occurred in utero^19^. The syncytiotrophoblasts are thought to be the main protagonist when it comes to transplacental infection. In fact, they are the barrier between the infections that are passed on from the mother to the fetus in vertical transmissions, not only regarding COVID-19. Controversy arises from the fact that the placenta is often infected, but the infection is not passed on to the fetus. This could suggest a “placental barrier”. Some early studies conducted SARS-CoV-2 cannot enter placental villi for a lack of caveolin. Further studies showed that the placenta expresses ACE2 and TMPRSS2 that may facilitate cell entry of the virus by binding to the viral spike protein^20^. In the setting of Boston Medical Center, a retrospective and prospective cohort study was conducted with a relatively small sample size from a single clinical site, to determine if the timing in which the maternal infection in pregnancy would change the outcome of the infection in the fetus. Samples were taken from the decidua basalis and analyzed for specific immune cell types dependent on gestational timing of SARS-CoV-2 infection. Results suggest that there is in fact an innate and adaptive immune response mounted at the placental level^16^. Varying levels of IL-6, IL-8, IL-10, and TNF-α are present, thought to be synthesized by maternal macrophages at the maternal-fetal interface. Infections during the second trimester showed downregulation of these cytokines suggesting a resolving immune response compared to infections in other trimesters. Likewise, for an infection to occur, there must be an interaction between the host and antigen. In this case it has been shown that mothers may be more susceptible to infections since pregnancy may be considered an immunosuppressed state. Other studies show that different comorbidities may aggravate the course of the infection in pregnant patients. SARS-CoV-2 has been found to have a higher rate of infection in pregnant women with gestational diabetes, hypertension, cardiovascular disease and asthma^21^.

## 5. Fetal and neonatal impact

Since pregnant women are more susceptible to COVID-related illness and hospitalizations, researchers initially believed the projected miscarriage rate for pregnant women would be 57% based on data from SARS and MERS outbreaks^18^. Initial reviews done on the outcome of pregnant women infected with SARS, MERS, and COVID-19 during pregnancy show increased preeclampsia, C-section, perinatal death, and miscarriage due to fetal distress^22^. This was caused by elevated levels of Il-6, IL-8, and TNF-α, increased hypercoagulation, and systemic inflammation leading to implantation failure and miscarriage^22^. Earlier systematic reviews also believed that widespread systemic inflammation would cause respiratory dyspnea, nervous system dysplasia, abnormal nervous and respiratory system development, and fetal demise^22^. Meta-analysis was used to analyze research done between December 1^st^, 2019, and March 31^st^, 2021, on earlier COVID maternal infections showed that overall, the miscarriage rate was similar between the general population and COVID-19 pregnant women. Miscarriages (<22 weeks) for COVID-19 pregnant women were 15.3% -23.1% with 95% CI, while the overall miscarriage (<20 weeks) rate for normal pregnant women was between 10-26%^23^. Contrarily to earlier studies done on COVID-19, there were no associations between COVID-19 infection and miscarriage, and SARS-CoV-2 infection during the first trimester had no impact on early pregnancy loss^23^. Initially, there were discrepancies between WHO, CDC, and AAP regarding protocols for mothers and neonates exposed to COVID-19. Studies completed during the past year have given more concise guidance on the management of these patients^24^. Research shows that although there were cases of horizontal transmission through respiratory droplets when newborns were exposed to infected caregivers, the overall risk of acquired newborn infection was low when infection precautions were implemented. Breastfeeding was encouraged since the transmission of IgA and IgG antibodies helped neutralize SARS-CoV-2 activity, which in turn could provide neonatal immunity^24^. In addition, the levels of IgG against the virus found only within the cord blood of seropositive women indicated that maternal antibody protection is possible^24^. In general, if test results are not yet available, it is safe to assume that neonates born to infected mothers are positive for SARS-CoV-2 and should be isolated from other healthy neonates^24^. Labor and delivery procedures as well as the type of birth (vaginal or C-section) should not be impacted by maternal infection status, and safety precautions should be used for all delivery personnel to ensure protection against maternal virus and aerosol spread^24^.

## **6. Perinatal **outcome

The normal physiologic changes that occur during pregnancy are like COVID symptoms; rhinitis, dyspnea, and changes in lung volume, making it harder to differentiate between the two diagnoses^25^. Overall, the cardiovascular and pulmonary changes that occur during pregnancy increase the potential for infection and hypoxia. During pregnancy, Th1 helper cells decrease while Th2 helper cells increase causing a decrease in cell-mediated immunity and increasing maternal predisposition to infection^25^. Influenza studies done on mouse colonies showed that pregnancy increased the Th1 inflammatory response pathway causing more physiological stress in the lungs. During COVID-19 infection, these same pregnant mouse colonies showed an increase in the Th2 pathway, activating an earlier protective immune response that leads to milder infections^25^.

The gold standard diagnosis for COVID-19 is RT-PCR which gives a quantitative value for the viral load. Although COVID assays provide faster results and are more widely available, they do report a low false-negative rate possibly due to undetected viral loads. It is difficult to expand the use of RT-PCR since it requires specific equipment and reagents, a colder temperature, and can only be done in a biosafety level 2 lab (BSL-2)^25^. Current pregnancy-specific treatment includes management of sepsis and respiratory distress which are common complications of COVID infection. For nonpregnant patients, higher sequential organ failure assessment (SOFA) scores and D-dimer (a small protein fragment found in the blood following fibrinolysis) levels >1 μg/mL on admission correlates with increased mortality. Using D-dimer levels to predict outcomes is difficult in pregnant patients since this value is generally higher and it is rare to find normal values within this population^25^. Changes in renal perfusion and the increase in oxygen demand to support placental perfusion should be considered when calculating SOFA scores to account for normal physiologic changes during pregnancy^25^. Per WHO guidelines, the use of systemic corticosteroids is not recommended since it delays viral clearance and has no impact on survival^26^.

There is conflicting information on predicting the course of SARS-CoV-2 infection in pregnant women and newborns. While some studies show fluctuations in the clinical presentation of symptoms, the consensus is that it is still not clear whether the infection causes pregnancy complications^27^. A study done at New-Jahra Hospital in Kuwait using retrospective chart data from 185 patients between March 15^th^, 2020, to May 31^st^, 2020, was used to analyze the clinical outcomes of pregnancy, maternal and fetal health for COVID-19 positive mothers^27^. Out of 185 women, 1.1% had severe pneumonia while most (88%) had mild symptoms- fever being the most common seen in 58% followed by cough seen in 50.65%, and rhinorrhea/sore throat in 24.3%. Out of the 185, 1.6% had a miscarriage, 0.54% had fetal demise (not due to COVID), and 89% had live births, leaving 8.6% with ongoing pregnancies at the time of analysis. The most common lab finding (42.1%) was elevated lactate dehydrogenase, followed by 37.1% with elevated C-reactive protein (CRP), 24.2% with elevated alanine transaminase, and 15.7% with lymphopenia^27^. The majority (97.3%) of patients who tested positive with PCR were given Oseltamivir, 24.3% received ceftriaxone, and 96.7% received Low Molecular Weight Heparin (LMWH). The patients with pneumonia were treated in the ICU without the use of extracorporeal membrane oxygenation and were eventually discharged^27^.

**Figure 2:**
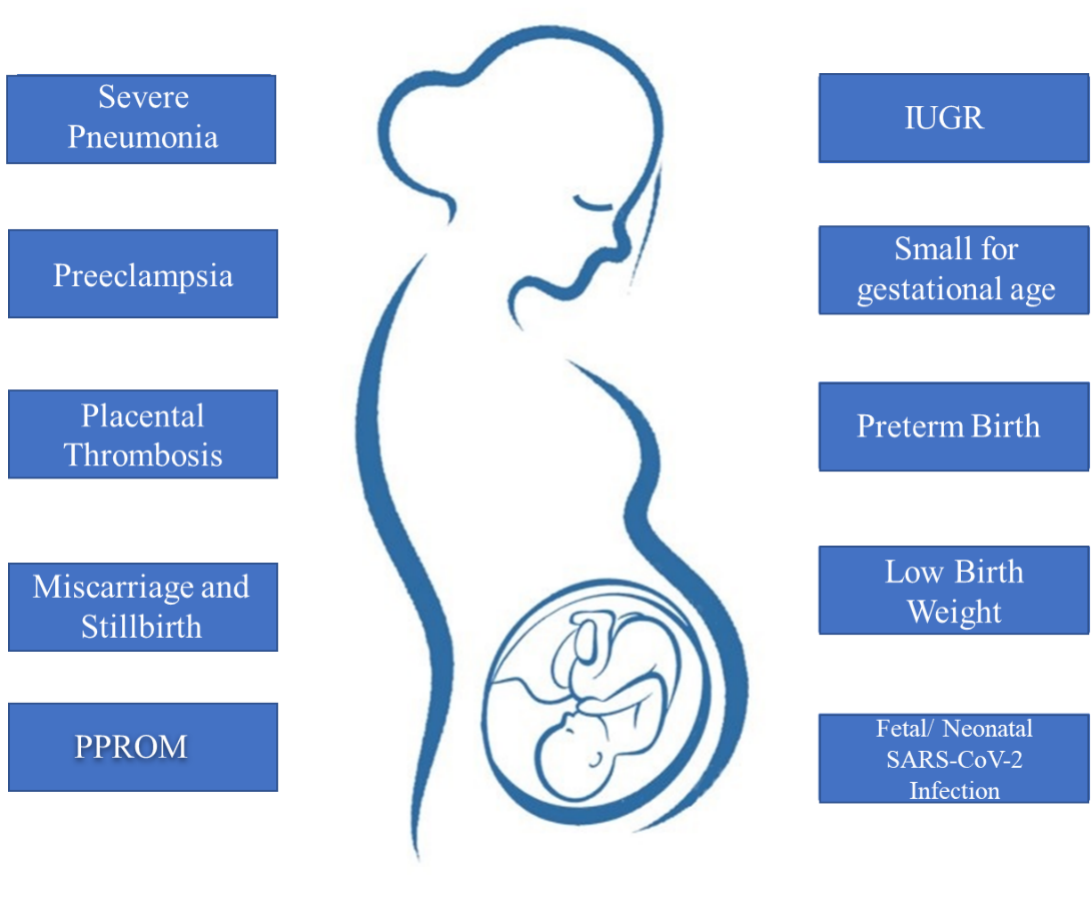
Impact of COVID-19 on pregnancy

Out of 165 deliveries, 26.6% were preterm and 47.8% (79) were born via cesarean section. 78 of the 79 C-sections were done because of fetal distress, and one was done because of fetal hypoxia caused by maternal pneumonia. While there were no neonatal deaths, 3% of neonates presented with hyaline membrane disease, and 1.2% tested positive for COVID. As well, COVID-19 infection in mother gives rise to risks of having small for gestational age (SGA) and low birth weight infants. Most preliminary studies noted fever and cough as most seen in COVID positive pregnant patients^27^. In addition, most studies showed that COVID infections occur most commonly during the 2nd and 3rd trimesters and have a positive correlation with gestational diabetes^28^. Since pregnancy naturally causes a hypercoagulable state, monitoring D-dimer levels is encouraged to prevent maternal mortality, especially when COVID infections are involved^27^. Overall, the ICU admission rates among COVID pregnant women were about the same as the healthy population and experienced positive outcomes for both their own health and the health of their newborns^27^. Figure 2 presents the effects of SARS-CoV-2 infection in pregnancy.

## 7. Role of vaccination

Maternal immunization is a public health strategy that tries to protect both the woman and her fetus or newborn infant from specific illnesses. Vaccination of pregnant women causes the production of vaccine-specific antibodies, which are then passed on to the children via the placenta or nursing^29^. Due to the efforts of the scientific community and collaboration between the federal government and the pharmaceutical industry, multiple viable and safe COVID-19 vaccines have been created at an unprecedented pace. However, because pregnant and breastfeeding women are still excluded from COVID antiviral and vaccination trials, there is a contradiction of a lack of empirical evidence in a high-risk population^30^.

The Food and Drug Administration (FDA) suggested undertaking developmental and reproductive toxicology studies before enrolling pregnant people or those who are not actively avoiding pregnancy in clinical trials, in acknowledgment of the necessity of including pregnant women in COVID-19 vaccination clinical trials. CDC has also developed a free smartphone app called "v-safe" that allows people, including pregnant women, to report adverse events after receiving the COVID-19 vaccine. Over 50,000 pregnant women have been studied so far, and no major vaccine-related side events have been reported. The United Kingdom has likewise established a comparable register for its inhabitants, with identical results and no safety issues with the COVID-19 vaccine^30^.

Despite the potentially fatal implications of COVID-19 infection in pregnant women and the availability of safe and effective (in nonpregnant populations) COVID-19 immunization, there is a paucity of published data on the safety and efficacy of any COVID-19 vaccine in human pregnancy. Shimabukuro et al. reviewed data from safety surveillance registries, such as “v-safe” and the Vaccine Adverse Event Reporting System (VAERS), on the safety of mRNA COVID-19 immunizations in pregnant women^31^. A total of 35,691 pregnant v-safe participants were enrolled and the most common local and systemic effects following immunization were injection-site pain, weariness, headache, and myalgia, which were more common after the second dose^30^.

Vaccination during pregnancy has been shown to protect both the mother and the baby from infectious diseases such as influenza and pertussis. COVID-19 vaccination based on mRNA nanoparticles and adenovirus vector, according to many expert committees, poses no major harm to pregnant or breastfeeding newborns. Shimabukuro et al. observed pregnancy loss in 13.9% of individuals who had completed pregnancy, based on an examination of safety surveillance registries such as “v-safe” and VAERS (i.e., live-born infant, spontaneous abortion, induced abortion, or stillbirth). They also mention preterm birth (9.4%) and small size for gestational age as negative neonatal outcomes (3.2%). There were no neonatal deaths reported. Many scientists believe that vaccine-stimulated immunoglobulin A can transfer through breast milk and provide further protection against COVID-19 to newborns^30^. Golan et al. found the presence of vaccine-derived IgA antibodies in breastmilk 3-4 weeks post-vaccination with mRNA COVID-19 vaccine (n=23). Moreover, discovered that the IgA antibody titers in the breastmilk of participants who received COVID-19 vaccination and COVID-19 infection were identical^32^.

COVID-19 vaccine is the most promising method of containing the global pandemic of COVID-19. It is critical to protect our vulnerable pregnant and nursing women while also emphasizing their participation in vaccination and antiviral therapy, clinical studies, and vaccine administration^30^.

## 8. Currently available treatment options, their efficacy and safety

Preferably, pregnant women with COVID-19 should be hospitalized irrespective of the severity of the disease^33^. General treatment remains the same for pregnant women and the general population. It includes (1) monitoring vitals, symptoms, FiO2, complete blood count, liver and renal function, CRP, and chest imaging, (2) oxygen therapy with achieving oxygen saturation over 95 percent, and (3) antibiotics for secondary bacterial infection (4) maintaining fluid and electrolyte balance^34^.

### **8.1 **Antivirals

Limited data is available for antiviral drugs used in pregnant women with COVID-19^35^. Below, some of the most useful ones are briefly described.

#### Lopinavir/Ritonavir (LPV/r)

Protease inhibitors are known for their use in the treatment of the Human Immunodeficiency Virus (HIV)^36^. It reduces mortality when used in the treatment of COVID-19 during pregnancy^36^. The potential adverse event includes hepatotoxicity, which exaggerates COVID-19-related liver dysfunction^37^. Roberts SS et al. conducted a study using an antiretroviral pregnancy registry to estimate birth defects due to exposure to LPV/R in HIV-positive pregnancies showing the safety profile of LPV/R used during pregnancy^38^. The dosage required is LPV/r (200mg/50mg), two capsules orally with alpha interferon (5 million IU in 2 mL of sterile water for injection), and nebulized inhalation twice a day^39^.

#### Remdesivir

It is a nucleoside analog, acting by inhibiting viral RNA-dependent RNA polymerase^40^. It is the first drug approved by FDA for the treatment of COVID-19 (October 22, 2020)^41^. In 2020, Burwick et al., through a remdesivir compassionate use program in pregnant women with severe COVID-19 showed increased recovery and decreased adverse events with a remdesivir treatment course of 10 days (200 mg on day 1 and 100 mg from 2-10 days, given intravenously) during pregnancy^42^. Contraindications included (1) glomerular filtration rate < 30 L/min, and (2) alanine aminotransferase level > 5 times the upper limit of normal^37^. Certain studies have identified remdesivir to be effective and safe during pregnancy, and its efficacy reached up to 68%^33^.

#### Molnupiravir

It is an FDA-approved Emergency use authorization (EUA) antiviral medication for the treatment of COVID-19^43^. However, molnupiravir is not authorized to be used during pregnancy due to its teratogenic potential, until further studies^43^.

#### Nirmatrelvir-Ritonavir (Paxlovid)

It is the first FDA-approved oral antiviral drug for EUA in the treatment of COVID-19 disease^44^. Paxlovid is a combination of nirmatrelvir, which stops the virus from replicating, and ritonavir, which prolongs its duration of action^45^. As Vitiello, Antonio, et al. in his review mentioned about embryo-fetal developmental toxicity studies in the rat or rabbit, (1) no teratogenic effects were seen except (2) reduction in fetal body weight in the rabbit^46^. So, it is not recommended to use during pregnancy until further clinical trials^46^.

#### Chloroquine and Hydroxychloroquine

The emergency use authorization of chloroquine and hydroxychloroquine for the treatment of COVID-19 was revoked by FDA on June 15, 2020^47^. These antimalarial drugs were used in the treatment of COVID-positive pregnancies due to their safety profile, especially with hydroxychloroquine compared to other antimalarials^48^. In 2020, Sisti et al. conducted a case report of a middle-aged woman G6P4014 at 26 weeks of gestation with COVID-19, treated with the combination of hydroxychloroquine (started on 400 mg on day 2 of hospitalization and 400 mg for the next 5 days, orally) and azithromycin (500 mg on day 1 of hospitalization and 250 mg for the next 4 days, orally) showing clinical improvement with low adverse outcomes except for QTc prolongation which got resolved with magnesium therapy^49^. Vincent et al. reported that chloroquine terminal glycosylation of the cellular receptor, angiotensin-converting enzyme 2 affected virus receptor binding, these inhibitory effects were present before and after exposure to the virus^50^. Thus, the authors concluded the potency of chloroquine in preventing and treating SARS-CoV-2^50^. Despite that, high dose chloroquine can cause systolic hypotension exaggerating supine hypotension in pregnancy due to aortocaval compression by the gravid uterus^33^. The recommended dose for hydroxychloroquine is 400mg, twice a day on day 1 followed by 200mg, twice a day from 2-4 days, given orally, and chloroquine 1 g on day 1 followed by 500mg from 2-7 days depending on recovery^37^.

### 8.2 Immunomodulatory Agents

#### Interferon-alpha

Interferon-alpha has also been suggested for use during pregnancy in women suffering from COVID-19^39^. Interferon-alpha inhibits viral synthesis through its immunomodulatory effects^51^. Certain studies have shown that a combination of interferon-alpha with lopinavir/ritonavir or favipiravir works effectively against SARS-CoV2 when administered via nebulization in a negative pressure room to prevent the dissemination of the virus through aerosol^51^. The recommended dosage is 5 million units twice per day^51^. The use of IFN-α during pregnancy is assumed to have abortifacient effects^51^. Therefore, IFN-α aerosol inhalation therapy should be used during pregnancy only if the benefits outweigh the potential risks to the fetus^51^.

#### Baricitinib (Olumiant)

The first immunomodulatory medication, baricitinib (Olumiant), was authorized by FDA on May 10, 2022, for the treatment of COVID-19^52^. It is the Janus Kinase (JAK) inhibitor, previously used in combination with remdesivir to treat COVID-19^53^. Animal embryo-fetal development studies in pregnant rats and rabbits at the maximum recommended human dose (MRHD) showed (1) reduced fetal body weight and skeletal malformations (2) embryo lethality in rabbits (3) No developmental toxicity during organogenesis at approximately 5 and 13 times MRHD respectively^54^. However, more clinical studies are required to establish the efficacy and safety profile of baricitinib use during COVID-19 pregnancy^54^.

#### Tocilizumab

It is an FDA-approved EUA medication for the treatment of COVID-19^55^. In 2020, Naqvi et al. mentioned a case report of 35-year-old primigravida at 22 weeks of pregnancy with COVID-19 treated with remdesivir (200mg on day 4 followed by 100 mg from 5-8 days of hospitalization, intravenously) and tocilizumab, and IL-6 monoclonal antibody (400mg, intravenously on day 3 of hospitalization), combination showing normalization of serum inflammatory markers (IL-6, C-reactive protein), decrease in oxygen requirement, and improvement in the clinical course of infection with less adverse events^56^.

#### Corticosteroids (prednisone, dexamethasone, hydrocortisone)

Corticosteroids are used commonly for fetal lung maturation (betamethasone- 12 mg, given intramuscularly 24 hours apart) during pregnancy^57^. Corticosteroids such as dexamethasone can be administered to COVID-19 pregnant patients with an increased risk of complications such as premature survival and the need for oxygen and mechanical ventilation^37^. However, the use of Dexamethasone during the 1st trimester and after 37 weeks of gestation is contraindicated^35^. In 2020, Zhou et al. performed a decision analysis in preterm prelabor rupture of membranes (PPROM) and COVID-19 positive pregnancies, showing the effectiveness of the administration of antenatal corticosteroids before 31 weeks of gestation as it increased maternal and infant quality-adjusted life years^57^. Despite the benefits, corticosteroids with their immunosuppression effects can cause worsening infection in critically ill patients^37^. The treatment course is of short duration with methylprednisolone 1-2 mg/kg/day^39^. High doses of dexamethasone should also be avoided during pregnancy due to its negative effects on the fetus^37^.

### 8.3 Neutralizing Antibodies

#### Bamlanivimab-Etesevimab/Casirivimab-Imdevimab (REGEN-COV)/Tixagevimab-Cilgavimab (Evsheld)

FDA revoked EUA for bamlanivimab (a monoclonal antibody that blocks the virus attachment and entry into human cells) in the treatment of COVID-19^58^. However, it gave emergency use authorization to bamlanivimab and etesevimab/casirivimab and imdevimab/tixagevimab, and cilgavimab, when used in combination for prevention against SARS CoV-2^59-61^. These antibodies work by preventing the binding of the spike protein of SARS-CoV-2, to its receptor on target host cells, thereby decreasing viral load^62^. In February 2022, Richley, Michael, et al. conducted a retrospective case series on monoclonal antibodies (bamlanivimab plus etesevimab or casirivimab plus imdevimab) use in pregnant persons with COVID-19 concluding favorable outcomes in pregnant women with its use^62^.

#### Sotrovimab

Its EUA for treatment of COVID-19 was revoked by FDA on April 5, 2022, due to its ineffectiveness against the Omicron subvariant^63^.

#### Bebtelovimab

On February 11, 2022, FDA authorized a EUA against the Omicron subvariant^64^. The infusion-related infections including anaphylaxis have been seen as adverse events with infusion of bebtelovimab^65^. It is not recommended during pregnancy unless potential benefits outweigh risks, despite cross-reactivity studies using human fetal tissue showing no significant binding of clinical concern^65^.

#### Convalescent Plasma Transfusion* (CPT)*

FDA has authorized high titer COVID-19 CPT for Emergency Use Authorization against SARS CoV-2^66^. Convalescent Plasma (CP) therapy includes using blood plasma from recently recovered COVID-19 patients containing antibodies targeting SARS-CoV-2^67^. In 2020, Grisolia et al. reported a case of a 29-year-old woman with G2P1 24 2/7 weeks of gestation with COVID-19 transfused with 300 mL of CP on day 7 and day 12 from the onset of symptoms resulting in (1) improvement in lymphocyte count (2) decrease in C-reactive protein and ferritin (3) decrease in oxygen requirement and resolution of inflammatory lung damage and acute respiratory distress syndrome (ARDS) (4) rapid recovery with no adverse outcomes^68^. Thus, the authors concluded an improved clinical response with CP therapy in COVID-19 positive pregnancies^68^. In 2021, Franchini et al. conducted a systemic review involving 12 case reports related to CP therapy administered between 21-36 (+/- 2) weeks of pregnancy with COVID-19 results showed that (1) two CP units at the first on day 2 of hospitalization showed clinical improvement in the majority of cases^69^. Moreover, CP therapy efficacy is greater when administered within 72 hours of hospitalization (2) one case showed neutralization of SARS CoV-2 (3) improved maternal and fetal outcomes with decreased maternal morbidity in the majority of cases (4) no adverse outcomes in the majority of cases proved the safety of CP therapy^69^. Thus, the authors concluded the efficacy and safety of CP therapy in COVID-positive pregnancies^69^. Despite that, CP therapy may cause infection with blood-borne pathogens, Hepatitis B Virus, Hepatitis C Virus, and HIV^67^. The treatment protocol is administrating CP in a single dose of 200 mL^37^.

### 8.4 **Thromboprophylaxis**

Pregnancy is a hypercoagulable state; infection with COVID-19 can exaggerate the risk for venous thromboembolism (VTE)^70,71^. According to The Royal College of Obstetricians and Gynecologists (RCOG), all pregnant women with COVID-19 should be assessed for VTE and receive prophylactic Low Molecular Weight Heparin (LMWH) unless birth is expected within 12 hours^70,71^. Prophylaxis with LMWH (4000 IU/day) requires to be continued through the puerperium until the patient is diagnosed with COVID-19^37^. Long-term adverse effects are heparin-induced thrombocytopenia and osteoporosis^37^. Table 1 summarizes currently available therapeutic options for the treatment of COVID-19 during pregnancy.

However, pregnant women with COVID-19 should be alert while using these drugs. Physicians should only prescribe these drugs if the benefits outweigh the risks in this population. Moreover, they should also be monitored for adverse effects and possible toxicity to decrease maternal-fetal morbidity and mortality^33^. Emphasis should be made to include pregnant women in clinical trials for the treatment of COVID-19 leading to the development of efficacious treatment with improved maternal and fetal outcomes in this population^72^.

**Table 1:** Table title Table caption

No.	Therapeutics option	Mechanism of action	Efficacy	Safety
Antivirals				
1	Lopinavir/Ritonavir (LPV/r)	Antiretroviral, protease inhibitors	Decreases mortality	Safe
2	Remdesivir	Nucleoside analog, inhibits SARS-CoV2 RNA- dependent RNA polymerase	Increases recovery and decreases adverse events	Insufficient data to determine its safety
3	Molnupiravir	Mutagenesis, interferes with viral replication	No data	No data
4	Nirmatrelvir-Ritonavir (Paxlovid)	Nirmatrelvir is a SAR-CoV-2 protease inhibitor whereas ritonavir prolongs its duration of action	No data	No data
5	Chloroquine and Hydroxychloroquine	Interferes with terminal glycosylation of ACE2, in vitro activity against SARS-CoV-2	FDA revoked its EUA against SARS-CoV-2	May lead to systolic hypotension with high dose chloroquine and QTc prolongation when hydroxychloroquine is combined with azithromycin
Immunomodulatory Agents				
6	Interferon-alpha	Immunoregulatory effects	Effective when used in combination with LPV/r	Insufficient data to determine its safety
7	Baricitinib (Olumiant)	JAK inhibitor, intracellularly inhibits the release of pro-inflammatory cytokines	No data	No data
8	Tocilizumab	IL-6 monoclonal antibody, inhibiting the pro-inflammatory activity of IL-6	Normalize inflammatory markers, decreases oxygen requirement, faster recovery, and decreases adverse events when used in combination with remdesivir	Insufficient data to determine its safety
9	Corticosteroids	Anti-inflammatory effects	Improves clinical course and decreases maternal and fetal mortality as well as morbidity	Safe, generally used for preterm lung maturation during pregnancy
Neutralizing Antibodies				
10	Bamlanivimab-Etesevimab/Casirivimab-Imdevimab (REGEN-COV)/Tixagevimab-Cilgavimab (EVUSHELD)	Prevents receptor binding of the spike protein of SARS-CoV2, decreases viral load	Favorable outcomes are seen in pregnancy	Insufficient data to determine its safety
11	Sotrovimab	FDA revoked its use, ineffective against Omicron sub-variant		
12	Bebtelovimab	Binds to spike protein of SARS CoV-2, decreases virus replication	Effective against Omicron sub-variant	Insufficient data to determine its safety
13	Convalescent Plasma Transfusion	High titer antibodies against SARS-CoV-2 from recently recovered patients	Increase in lymphocyte count, decrease in inflammatory markers, decrease in oxygen requirement, and decrease in maternal mortality	Insufficient data to determine its safety
Thromboprophylaxis				
14	Low Molecular Weight Heparin	Anti-coagulant properties	Prevents venous thromboembolism (VTE)	Safe

## **9. Labor **and delivery guidance

During the COVID-19 pandemic, labor and delivery come with its own unique guidelines. The hospital admission for these patients is planned and therefore patients are required to follow certain protocols to protect the health of the mother and the baby. The recommendations are as follows:

### 1. Take off from work 2 weeks prior to the date of delivery and maintain strict isolation^72^.

#### 2. Screen the patient and her partner a day before the planned date of admission^72^.

#### 3. Labor and delivery triage patients and partners undergo verbal screening for upper respiratory infection symptoms^73^.

a. If patients are positive for symptoms, provide a mask and further evaluate by the obstetric provider^73^.

b. If the partner is positive for symptoms, refer to a medical care provider^73^.

c. If a patient is COVID-19 positive, personal protective equipment should be utilized including a surgical mask and/or N95 mask, protective eyewear, gloves, and gown. Practice strict hand hygiene^72^.

d. Precautions should be taken to avoid the use of aerosolized oxygen^73^.

#### 4. Visitor policy: one appointed adult should be allowed to visit and be easily identified using a colored wristband. This support person should be designated for the entire admission^72^.

#### 5. Recommendations of labor induction vary with current protocols of the region delivery is taking place^72^.

a. During the first stage of labor, general management should occur as usual, including necessary intrapartum antibiotic use and oxytocin^73^.To reduce the risk of COVID-19 spread, oxygen use in fetal resuscitation and nitrous oxide use are not recommended^74,75^_,_

b. The use of anesthesia during COVID-19 is not contraindicated^75^. Regional anesthesia is encouraged since it reduces maternal respiratory distress due to pain and anxiety during labor^76^.

#### 6. Care in labor:

a. Aim to keep oxygen saturation >94% and titrate accordingly^77^.

b. If women have signs of sepsis, investigate and treat as per guidance on sepsis in pregnancy but also consider active COVID-19 as the cause^77^.

c. The mode of birth should not be influenced by COVID-19 unless the woman's respiratory condition demands urgent delivery^77^.

d. When cesarean birth or other operative procedure is advised it should be done after wearing PPE^77^.

#### 7. Fetal monitoring and procedures:

a. Continuous electronic fetal monitoring is recommended for all symptomatic patients^78^.

b. COVID-19 is not a contraindication to rupture of fetal membranes, application of a fetal scalp electrode, or insertion of intrauterine pressure catheter^78^.

#### 8. Management of the second stage:

a. Pushing should not be delayed since this involves repeated forceful exhalation and loss of feces which can increase the risk of virus transmission^78^.

b. Patients who are positive for COVID-19 should wear a mask but if they are uncomfortable during exertion, they can remove it^78^.

c. Delayed umbilical cord clamping in patients is highly unlikely to increase the risk of vertical transmission^78^.

#### 9. Management of the third stage:

a. Patients who develop postpartum hemorrhage can be managed according to standard protocols^78^.

b. Mothers with COVID-19 can safely practice skin-to-skin contact and breastfeed in the birthing room if they wear a surgical mask and use proper hand hygiene^78^.

c. No consensus regarding whether maternal COVID-19 is an indication for placental examination by a pathologist^78^.

#### 10. Medications in Obstetric COVID-19:

a. Indomethacin: this popular NSAID is thought to worsen the course of COVID-19 since the NSAID increases the expression of ACE2^79^. However, this has not been established, and several agencies, including WHO and FDA, have suggested that use of NSAID should not be restricted. Nifedipine may be explored as an option in the case of tocolysis^73^.

b. Betamethasone/Dexamethasone use should be limited after 34 weeks of gestation as there is an increased risk of COVID-19 related mortality associated with the use of steroids^72,79^.

c. Magnesium sulfate: may be used as indicated in patients with mild to moderate respiratory symptoms and in those with delivery before 32 weeks of gestation or preeclampsia^80^.

d. Recommendations on medication may change upon update to data^72^.

#### 11. Intrapartum care of COVID-19 positive patients:

Prioritize delivery of term COVID-19 positive patients with mild symptoms. To prevent transmission, a section of the ward should be selected for COVID-19 positive patients^72^. Postpartum care includes the expedited discharge of patients with normal vaginal delivery, patients discharged on day 1, and cesarean deliveries discharged on day 2. Telehealth should be used for consultation including wound healing, mastitis, and any additional routine concerns the family may have^72^. No evidence of transmission of COVID-19 via breast milk, hence, CDC recommends breast milk to infants especially due to the presence of protective antibodies^81^.

#### 12. Rooming-in for mother and baby:

Newborns should not be separated from their mother, regardless of COVID-19 status unless in the case of severe maternal or newborn illness^82^.

## 10. Conclusion

COVID-19 pandemic hit almost all aspects of people’s lives, its effect on pregnancy is also of serious concern. Pregnant women who are infected with COVID-19 are more prone to have an acute illness and it can result in several pregnancy complications like preterm labor and stillbirth. A rare number of newborns have been identified as COVID-19 positive although it is not certain when they got infected with the virus: prior to, during, or following birth. There have been little to no symptoms, and they recovered shortly after. The vaccines are proved to be effective against COVID-19 infection preventing both the mother and the child from getting seriously ill. A vaccinated mother can transfer antibodies to the baby against COVID-19 through breastfeeding, therefore, breastfeeding is recommended. For a safer and healthier pregnancy and childbirth, the recommended guidelines should be properly followed. Researchers are still investigating the additional effects of COVID-19 infection on pregnancy; we hope new findings will help us give more protection to the mother and child against this abysmal virus.

## References

[1] Chowdhury Selia, Bappy Mehedi Hasan, Chowdhury Samia, Chowdhury Md. Shahraj, Chowdhury Nurjahan Shipa (2021). Current Review of Delta Variant of SARS-CoV-2. European Journal of Medical and Health Sciences.

[2] Chowdhury Selia, Bappy Mehedi Hasan (2021). On the Delta Plus Variant of SARS-CoV-2. European Journal of Medical and Health Sciences.

[3] Chowdhury Selia, Bappy Mehedi Hasan, Chowdhury Samia, Chowdhury Md. Shahraj, Chowdhury Nurjahan Shipa (2021). COVID-19 Induced Cardiovascular Complications and Recent Therapeutic Advances. European Journal of Medical and Health Sciences.

[4] Chowdhury Selia, Bappy Mehedi Hasan, Chowdhury Samia, Chowdhury Md. Shahraj, Chowdhury Nurjahan Shipa (2022). Omicron Variant (B.1.1.529) of SARS-CoV-2, A Worldwide Public Health Emergency!. European Journal of Clinical Medicine.

[5] Arthurs Anya Lara, Jankovic-Karasoulos Tanja, Roberts Claire Trelford (2021). COVID-19 in pregnancy: What we know from the first year of the pandemic.. Biochimica et biophysica acta. Molecular basis of disease.

[6] Ortiz Edgar Iván, Herrera Enrique, De La Torre Alejandro (2020). Coronavirus (COVID 19) Infection in Pregnancy.. Colombia medica (Cali, Colombia).

[7] Wastnedge Elizabeth A. N., Reynolds Rebecca M., van Boeckel Sara R., Stock Sarah J., Denison Fiona C., Maybin Jacqueline A., Critchley Hilary O. D. (2021). Pregnancy and COVID-19. Physiological Reviews.

[8] Figueiro-Filho Ernesto Antonio, Hobson Sebastian R, Farine Dan, Yudin Mark H (2021). Highly expressed ACE-2 receptors during pregnancy: A protective factor for SARS-COV-2 infection?. Medical hypotheses.

[9] Bouachba Amine, Allias Fabienne, Nadaud Beatrice, Massardier Jerome, Mekki Yahia, Bouscambert Duchamp Maude, Fourniere Benoit De LA, Huissoud Cyril, Trecourt Alexis, Collardeau-Frachon Sophie (2021). Placental lesions and SARS-Cov-2 infection: Diffuse placenta damage associated to poor fetal outcome.. Placenta.

[10] Li Qi, Wang Weinan, Pei Chenlin, Zhao Yanhua, Liu Rong, Zhang Weishe, Huang Lihui, Li Tieping, Huang Jingrui (2021). Expression of SARS-CoV-2 entry genes ACE2 and TMPRSS2 at single cell resolution in the peripartum decidua.. American journal of translational research.

[11] Garrido-Pontnou Marta, Navarro Alexandra, Camacho Jessica, Crispi Fàtima, Alguacil-Guillén Marina, Moreno-Baró Anna, Hernandez-Losa Javier, Sesé Marta, Ramón Y Cajal Santiago, Garcia Ruíz Itziar, Serrano Berta, Garcia-Aguilar Paula, Suy Anna, Ferreres Joan Carles, Nadal Alfons (2021). Diffuse trophoblast damage is the hallmark of SARS-CoV-2-associated fetal demise.. Modern pathology : an official journal of the United States and Canadian Academy of Pathology, Inc.

[12] Schwartz David A, Baldewijns Marcella, Benachi Alexandra, Bugatti Mattia, Collins Rebecca R J, De Luca Danièle, Facchetti Fabio, Linn Rebecca L, Marcelis Lukas, Morotti Denise, Morotti Raffaella, Parks W Tony, Patanè Luisa, Prevot Sophie, Pulinx Bianca, Rajaram Veena, Strybol David, Thomas Kristen, Vivanti Alexandre J (2021). Chronic Histiocytic Intervillositis With Trophoblast Necrosis Is a Risk Factor Associated With Placental Infection From Coronavirus Disease 2019 (COVID-19) and Intrauterine Maternal-Fetal Severe Acute Respiratory Syndrome Coronavirus 2 (SARS-CoV-2) Transmission in Live-Born and Stillborn Infants.. Archives of pathology and laboratory medicine.

[13] Husen Marjolein F, van der Meeren Lotte E, Verdijk Robert M, Fraaij Pieter L A, van der Eijk Annemiek A, Koopmans Marion P G, Freeman Liv, Bogers Hein, Trietsch Marjolijn D, Reiss Irwin K M, DeKoninck Philip L J, Schoenmakers Sam (2021). Unique Severe COVID-19 Placental Signature Independent of Severity of Clinical Maternal Symptoms.. Viruses.

[14] Schwartz David A., Morotti Denise (2020). Placental Pathology of COVID-19 with and without Fetal and Neonatal Infection: Trophoblast Necrosis and Chronic Histiocytic Intervillositis as Risk Factors for Transplacental Transmission of SARS-CoV-2. Viruses.

[15] Sharps Megan C, Hayes Dexter J L, Lee Stacey, Zou Zhiyong, Brady Chloe A, Almoghrabi Yousef, Kerby Alan, Tamber Kajal K, Jones Carolyn J, Adams Waldorf Kristina M, Heazell Alexander E P (2020). A structured review of placental morphology and histopathological lesions associated with SARS-CoV-2 infection.. Placenta.

[16] Juttukonda Lillian J, Wachman Elisha M, Boateng Jeffery, Jain Mayuri, Benarroch Yoel, Taglauer Elizabeth S (2022). Decidual immune response following COVID-19 during pregnancy varies by timing of maternal SARS-CoV-2 infection.. Journal of reproductive immunology.

[17] Peng Zhoujie, Zhang Jing, Shi Yuan, Yi Ming (2022). Research progresses in vertical transmission of SARS-CoV-2 among infants born to mothers with COVID-19.. Future virology.

[18] Dong Lan, Tian Jinhua, He Songming, Zhu Chuchao, Wang Jian, Liu Chen, Yang Jing (2020). Possible Vertical Transmission of SARS-CoV-2 From an Infected Mother to Her Newborn.. JAMA.

[19] Novoa Rommy H, Quintana Willy, Llancarí Pedro, Urbina-Quispe Katherine, Guevara-Ríos Enrique, Ventura Walter (2021). Maternal clinical characteristics and perinatal outcomes among pregnant women with coronavirus disease 2019. A systematic review. Travel medicine and infectious disease.

[20] Rad Habib Sadeghi, Röhl Joan, Stylianou Nataly, Allenby Mark C, Bazaz Sajad Razavi, Warkiani Majid E, Guimaraes Fernando S F, Clifton Vicki L, Kulasinghe Arutha (2021). The Effects of COVID-19 on the Placenta During Pregnancy.. Frontiers in immunology.

[21] Diriba Kuma, Awulachew Ephrem, Getu Eyob (2020). The effect of coronavirus infection (SARS-CoV-2, MERS-CoV, and SARS-CoV) during pregnancy and the possibility of vertical maternal-fetal transmission: a systematic review and meta-analysis.. European journal of medical research.

[22] Favre Guillaume, Pomar Léo, Musso Didier, Baud David (2020). 2019-nCoV epidemic: what about pregnancies?. Lancet (London, England).

[23] Dang Dan, Wang Liying, Zhang Chuan, Li Zhenyu, Wu Hui (2020). Potential effects of SARS-CoV-2 infection during pregnancy on fetuses and newborns are worthy of attention.. The journal of obstetrics and gynaecology research.

[24] Cavalcante Marcelo Borges, de Melo Bezerra Cavalcante Candice Torres, Cavalcante Ana Nery Melo, Sarno Manoel, Barini Ricardo, Kwak-Kim Joanne (2021). COVID-19 and miscarriage: From immunopathological mechanisms to actual clinical evidence.. Journal of reproductive immunology.

[25] Shah Malika D, Saugstad Ola Didrik (2021). Newborns at risk of Covid-19 - lessons from the last year.. Journal of perinatal medicine.

[26] Dashraath Pradip, Wong Jing Lin Jeslyn, Lim Mei Xian Karen, Lim Li Min, Li Sarah, Biswas Arijit, Choolani Mahesh, Mattar Citra, Su Lin Lin (2020). Coronavirus disease 2019 (COVID-19) pandemic and pregnancy.. American journal of obstetrics and gynecology.

[27] (2020). Novel coronavirus technical guidance: patient management. World Health Organization (WHO).

[28] Ayed Amal, Embaireeg Alia, Benawadh Asmaa, Al-Fouzan Wadha, Hammoud Majdeda, Al-Hathal Monif, Alzaydai Abeer, Ahmad Ashraf, Ayed Mariam (2020). Maternal and perinatal characteristics and outcomes of pregnancies complicated with COVID-19 in Kuwait.. BMC pregnancy and childbirth.

[29] Knight Marian, Bunch Kathryn, Vousden Nicola, Morris Edward, Simpson Nigel, Gale Chris, O'Brien Patrick, Quigley Maria, Brocklehurst Peter, Kurinczuk Jennifer J (2020). Characteristics and outcomes of pregnant women admitted to hospital with confirmed SARS-CoV-2 infection in UK: national population based cohort study.. BMJ (Clinical research ed.).

[30] Sebghati Mercede, Khalil Asma (2021). Uptake of vaccination in pregnancy.. Best practice and research. Clinical obstetrics and gynaecology.

[31] Garg Ishan, Shekhar Rahul, Sheikh Abu B, Pal Suman (2021). COVID-19 Vaccine in Pregnant and Lactating Women: A Review of Existing Evidence and Practice Guidelines.. Infectious disease reports.

[32] Shimabukuro Tom T, Kim Shin Y, Myers Tanya R, Moro Pedro L, Oduyebo Titilope, Panagiotakopoulos Lakshmi, Marquez Paige L, Olson Christine K, Liu Ruiling, Chang Karen T, Ellington Sascha R, Burkel Veronica K, Smoots Ashley N, Green Caitlin J, Licata Charles, Zhang Bicheng C, Alimchandani Meghna, Mba-Jonas Adamma, Martin Stacey W, Gee Julianne M, Meaney-Delman Dana M (2021). Preliminary Findings of mRNA Covid-19 Vaccine Safety in Pregnant Persons.. The New England journal of medicine.

[33] Wu Di, Fang Dong, Wang Renjie, Deng Dongrui, Liao Shujie (2021). Management of Pregnancy during the COVID-19 Pandemic.. Global challenges (Hoboken, NJ).

[34] Rajewska Aleksandra, Mikołajek-Bedner Wioletta, Lebdowicz-Knul Joanna, Sokołowska Małgorzata, Kwiatkowski Sebastian, Torbé Andrzej (2020). COVID-19 and pregnancy - where are we now? A review.. Journal of perinatal medicine.

[35] Wang Chiu-Lin, Liu Yi-Yin, Wu Chin-Hu, Wang Chun-Yu, Wang Chun-Hung, Long Cheng-Yu (2021). Impact of COVID-19 on Pregnancy.. International journal of medical sciences.

[36] Narang Kavita, Enninga Elizabeth Ann L, Gunaratne Madugodaralalage D S K, Ibirogba Eniola R, Trad Ayssa Teles A, Elrefaei Amro, Theiler Regan N, Ruano Rodrigo, Szymanski Linda M, Chakraborty Rana, Garovic Vesna D (2020). SARS-CoV-2 Infection and COVID-19 During Pregnancy: A Multidisciplinary Review.. Mayo Clinic proceedings.

[37] Favilli Alessandro, Mattei Gentili Marta, Raspa Francesca, Giardina Irene, Parazzini Fabio, Vitagliano Amerigo, Borisova Anna V, Gerli Sandro (2022). Effectiveness and safety of available treatments for COVID-19 during pregnancy: a critical review.. The journal of maternal-fetal and neonatal medicine : the official journal of the European Association of Perinatal Medicine, the Federation of Asia and Oceania Perinatal Societies, the International Society of Perinatal Obstetricians.

[38] Roberts Susan S, Martinez Marisol, Covington Deborah L, Rode Richard A, Pasley Mary V, Woodward William C (2009). Lopinavir/Ritonavir in Pregnancy. JAIDS Journal of Acquired Immune Deficiency Syndromes.

[39] Liang Huan, Acharya Ganesh (2020). Novel corona virus disease (COVID-19) in pregnancy: What clinical recommendations to follow?. Acta obstetricia et gynecologica Scandinavica.

[40] Hayakawa Satoshi, Komine-Aizawa Shihoko, Mor Gil G (2020). Covid-19 pandemic and pregnancy.. The journal of obstetrics and gynaecology research.

[41] (2020). FDA approves first treatment for COVID-19. U.S. Food and Drug Administration; Accessed May 31, 2022..

[42] Burwick Richard M, Yawetz Sigal, Stephenson Kathryn E, Collier Ai-Ris Y, Sen Pritha, Blackburn Brian G, Kojic E Milunka, Hirshberg Adi, Suarez Jose F, Sobieszczyk Magdalena E, Marks Kristen M, Mazur Shawn, Big Cecilia, Manuel Oriol, Morlin Gregory, Rose Suzanne J, Naqvi Mariam, Goldfarb Ilona T, DeZure Adam, Telep Laura, Tan Susanna K, Zhao Yang, Hahambis Tom, Hindman Jason, Chokkalingam Anand P, Carter Christoph, Das Moupali, Osinusi Anu O, Brainard Diana M, Varughese Tilly A, Kovalenko Olga, Sims Matthew D, Desai Samit, Swamy Geeta, Sheffield Jeanne S, Zash Rebecca, Short William R (2020). Compassionate Use of Remdesivir in Pregnant Women With Severe Coronavirus Disease 2019. Clinical Infectious Diseases.

[43] Singh Awadhesh Kumar, Singh Akriti, Singh Ritu, Misra Anoop (2022). An updated practical guideline on use of molnupiravir and comparison with agents having emergency use authorization for treatment of COVID-19. Diabetes; Metabolic Syndrome: Clinical Research Reviews.

[44] (2021). Coronavirus (COVID-19) update: FDA authorizes first oral antiviral for treatment of COVID-19. U.S. Food and Drug Administration; Accessed May 31, 2022..

[45] Chaplin Steve (2022). Paxlovid: antiviral combination for the treatment of
<scp>COVID</scp>
‐19. Prescriber.

[46] Vitiello Antonio, Ferrara Francesco, Zovi Andrea, Trama Ugo, Boccellino Mariarosaria (2022). Pregnancy and COVID-19, focus on vaccine and pharmacological treatment. Journal of Reproductive Immunology.

[47] (2020). FDA cautions against use of hydroxychloroquine or chloroquine for COVID-19 outside of the hospital setting or a clinical trial due to risk of heart rhythm problems.. U.S. Food and Drug Administration; Accessed May 31, 2022..

[48] Kaplan Yusuf, Koren Gideon (2020). Use of hydroxychloroquine during pregnancy and breastfeeding: An update for the recent coronavirus pandemic (COVID-19). Motherisk Int J.

[49] Sisti Giovanni, Schiattarella Antonio, Sisti Andrea (2020). Treatment of COVID-19 in Pregnancy with Hydroxychloroquine and Azithromycin: a case report.. Acta bio-medica : Atenei Parmensis.

[50] Vincent Martin J, Bergeron Eric, Benjannet Suzanne, Erickson Bobbie R, Rollin Pierre E, Ksiazek Thomas G, Seidah Nabil G, Nichol Stuart T (2005). Chloroquine is a potent inhibitor of SARS coronavirus infection and spread.. Virology journal.

[51] Li Lu, Wang Xiaojuan, Wang Rongrong, Hu Yunzhen, Jiang Saiping, Lu Xiaoyang (2020). Antiviral Agent Therapy Optimization in Special Populations of COVID-19 Patients.. Drug design, development and therapy.

[52] (2022). FDA roundup: May 10, 2022. U.S. Food and Drug Administration; Accessed May 31, 2022..

[53] Kalil Andre C, Patterson Thomas F, Mehta Aneesh K, Tomashek Kay M, Wolfe Cameron R, Ghazaryan Varduhi, Marconi Vincent C, Ruiz-Palacios Guillermo M, Hsieh Lanny, Kline Susan, Tapson Victor, Iovine Nicole M, Jain Mamta K, Sweeney Daniel A, El Sahly Hana M, Branche Angela R, Regalado Pineda Justino, Lye David C, Sandkovsky Uriel, Luetkemeyer Anne F, Cohen Stuart H, Finberg Robert W, Jackson Patrick E H, Taiwo Babafemi, Paules Catharine I, Arguinchona Henry, Erdmann Nathaniel, Ahuja Neera, Frank Maria, Oh Myoung-Don, Kim Eu-Suk, Tan Seow Y, Mularski Richard A, Nielsen Henrik, Ponce Philip O, Taylor Barbara S, Larson LuAnn, Rouphael Nadine G, Saklawi Youssef, Cantos Valeria D, Ko Emily R, Engemann John J, Amin Alpesh N, Watanabe Miki, Billings Joanne, Elie Marie-Carmelle, Davey Richard T, Burgess Timothy H, Ferreira Jennifer, Green Michelle, Makowski Mat, Cardoso Anabela, de Bono Stephanie, Bonnett Tyler, Proschan Michael, Deye Gregory A, Dempsey Walla, Nayak Seema U, Dodd Lori E, Beigel John H (2021). Baricitinib plus Remdesivir for Hospitalized Adults with Covid-19.. The New England journal of medicine.

[54] (2018). OLUMIANT (baricitinib). U.S. Food and Drug Administration; Accessed May 31, 2022.

[55] (2021). Coronavirus (COVID-19) Update: FDA Authorizes Drug for Treatment of COVID-19. U.S. Food and Drug Administration; Accessed May 31, 2022.

[56] Naqvi Mariam, Zakowski Phillip, Glucksman Lindsey, Smithson Sarah, Burwick Richard M (2020). Tocilizumab and Remdesivir in a Pregnant Patient With Coronavirus Disease 2019 (COVID-19).. Obstetrics and gynecology.

[57] Zhou Clarice G, Packer Claire H, Hersh Alyssa R, Caughey Aaron B (2022). Antenatal corticosteroids for pregnant women with COVID-19 infection and preterm prelabor rupture of membranes: a decision analysis.. The journal of maternal-fetal and neonatal medicine: the official journal of the European Association of Perinatal Medicine, the Federation of Asia and Oceania Perinatal Societies, the International Society of Perinatal Obstetricians.

[58] (2021). Coronavirus (COVID-19) update: FDA authorizes drug for treatment of COVID-19.. U.S. Food and Drug Administration; Accessed May 31, 2022.

[59] (2021). FDA authorizes bamlanivimab and etesevimab monoclonal antibody therapy for post-exposure prophylaxis (prevention) for COVID-19.. U.S. Food and Drug Administration; Accessed May 31, 2022..

[60] Ning Lin, Abagna Hamza B, Jiang Qianhu, Liu Siqi, Huang Jian (2021). Development and application of therapeutic antibodies against COVID-19.. International journal of biological sciences.

[61] Fitzsimmons William E. (2022). COVID-19 vaccine associated transverse myelitis-Evusheld as an option when vaccination is not recommended due to severe adverse events. Human Vaccines; Immunotherapeutics.

[62] Richley Michael, Rao Rashmi R., Afshar Yalda, Mei Jenny, Mok Thalia, Vijayan Tara, Weinstein Stacey, Pham Christine U., Madamba Jason, Shin Christina S., Suda Deborah, Han Christina S. (2022). Neutralizing Monoclonal Antibodies for Coronavirus Disease 2019 (COVID-19) in Pregnancy. Obstetrics and Gynecology.

[63] (2022). FDA updates Sotrovimab emergency use authorization. U.S. Food and Drug Administration; Accessed May 31, 2022..

[64] Westendorf Kathryn, Žentelis Stefanie, Wang Lingshu, Foster Denisa, Vaillancourt Peter, Wiggin Matthew, Lovett Erica, van der Lee Robin, Hendle Jörg, Pustilnik Anna, Sauder J. Michael, Kraft Lucas, Hwang Yuri, Siegel Robert W., Chen Jinbiao, Heinz Beverly A., Higgs Richard E., Kallewaard Nicole L., Jepson Kevin, Goya Rodrigo, Smith Maia A., Collins David W., Pellacani Davide, Xiang Ping, de Puyraimond Valentine, Ricicova Marketa, Devorkin Lindsay, Pritchard Caitlin, O’Neill Aoise, Dalal Kush, Panwar Pankaj, Dhupar Harveer, Garces Fabian A., Cohen Courtney A., Dye John M., Huie Kathleen E., Badger Catherine V., Kobasa Darwyn, Audet Jonathan, Freitas Joshua J., Hassanali Saleema, Hughes Ina, Munoz Luis, Palma Holly C., Ramamurthy Bharathi, Cross Robert W., Geisbert Thomas W., Menachery Vineet, Lokugamage Kumari, Borisevich Viktoriya, Lanz Iliana, Anderson Lisa, Sipahimalani Payal, Corbett Kizzmekia S., Yang Eun Sung, Zhang Yi, Shi Wei, Zhou Tongqing, Choe Misook, Misasi John, Kwong Peter D., Sullivan Nancy J., Graham Barney S., Fernandez Tara L., Hansen Carl L., Falconer Ester, Mascola John R., Jones Bryan E., Barnhart Bryan C. (2022). LY-CoV1404 (bebtelovimab) potently neutralizes SARS-CoV-2 variants. Cell Reports.

[65] (2022). Emergency Use Authorization for Bebtelovimab Highlights of Emergency Use Authorization (EUA).. U.S. Food and Drug Administration; Accessed May 31, 2022; Accessed May 31, 2022..

[66] (2022). Recommendations for investigational COVID-19 convalescent plasma. U.S. Food and Drug Administration; Accessed May 31, 2022..

[67] Abd El-Aziz Tarek Mohamed, Stockand James D. (2020). Recent progress and challenges in drug development against COVID-19 coronavirus (SARS-CoV-2) - an update on the status. Infection, Genetics and Evolution.

[68] Grisolia Gianpaolo, Franchini Massimo, Glingani Claudia, Inglese Francesco, Garuti Martina, Beccaria Massimiliano, Capuzzo Martina, Pinto Alessia, Pavan Giorgia, Righetto Lara, Perotti Cesare, Zampriolo Paolo, De Donno Giuseppe (2020). Convalescent plasma for coronavirus disease 2019 in pregnancy: a case report and review.. American journal of obstetrics and gynecology MFM.

[69] Franchini Massimo, Prefumo Federico, Grisolia Gianpaolo, Bergamini Valentino, Glingani Claudia, Pisello Marlene, Presti Francesca, Zaffanello Marco (2021). Convalescent Plasma for Pregnant Women with COVID-19: A Systematic Literature Review.. Viruses.

[70] Khoiwal Kavita, Kapur Dhriti, Gaurav Amrita, Chaturvedi Jaya (2020). Management of Pregnant Women in Times of Covid-19: A Review of Current Literature.. Journal of obstetrics and gynaecology of India.

[71] Ryan Gillian A, Purandare Nikhil C, McAuliffe Fionnuala M, Hod Moshe, Purandare Chittaranjan N (2020). Clinical update on COVID-19 in pregnancy: A review article.. The journal of obstetrics and gynaecology research.

[72] Taylor Melanie M, Kobeissi Loulou, Kim Caron, Amin Avni, Thorson Anna E, Bellare Nita B, Brizuela Vanessa, Bonet Mercedes, Kara Edna, Thwin Soe Soe, Kuganantham Hamsadvani, Ali Moazzam, Oladapo Olufemi T, Broutet Nathalie (2021). Inclusion of pregnant women in COVID-19 treatment trials: a review and global call to action.. The Lancet. Global health.

[73] Boelig Rupsa C, Manuck Tracy, Oliver Emily A, Di Mascio Daniele, Saccone Gabriele, Bellussi Federica, Berghella Vincenzo (2020). Labor and delivery guidance for COVID-19.. American journal of obstetrics and gynecology MFM.

[74] Interim infection prevention and control recommendations for patients with suspected or confirmed coronavirus disease 2019 (COVID-19) in healthcare settings.. Centers for Disease Control and Prevention (CDC).

[75] Hamel Maureen S, Anderson Brenna L, Rouse Dwight J (2014). Oxygen for intrauterine resuscitation: of unproved benefit and potentially harmful.. American journal of obstetrics and gynecology.

[76] Pountoukidou Argyro, Potamiti-Komi Maria, Sarri Vrisiis, Papapanou Michail, Routsi Eleni, Tsiatsiani Anna Maria, Vlahos Nikolaos, Siristatidis Charalampos (2021). Management and Prevention of COVID-19 in Pregnancy and Pandemic Obstetric Care: A Review of Current Practices.. Healthcare (Basel, Switzerland).

[77] (2022). Guidance for Management of Pregnant Women in COVID-19 Pandemic. ICMR.gov.in.

[78] (2022). COVID-19: Intrapartum and postpartum issues. UpToDate. Uptodate.com.

[79] Fang Lei, Karakiulakis George, Roth Michael (2020). Are patients with hypertension and diabetes mellitus at increased risk for COVID-19 infection?. The Lancet. Respiratory medicine.

[80] Zhou Fei, Yu Ting, Du Ronghui, Fan Guohui, Liu Ying, Liu Zhibo, Xiang Jie, Wang Yeming, Song Bin, Gu Xiaoying, Guan Lulu, Wei Yuan, Li Hui, Wu Xudong, Xu Jiuyang, Tu Shengjin, Zhang Yi, Chen Hua, Cao Bin (2020). Clinical course and risk factors for mortality of adult inpatients with COVID-19 in Wuhan, China: a retrospective cohort study.. Lancet (London, England).

[81] Chen Huijun, Guo Juanjuan, Wang Chen, Luo Fan, Yu Xuechen, Zhang Wei, Li Jiafu, Zhao Dongchi, Xu Dan, Gong Qing, Liao Jing, Yang Huixia, Hou Wei, Zhang Yuanzhen (2020). Clinical characteristics and intrauterine vertical transmission potential of COVID-19 infection in nine pregnant women: a retrospective review of medical records.. Lancet (London, England).

[82] Pavlidis Pollyanna, Eddy Katherine, Phung Laura, Farrington Elise, Connolly Mairead, Lopes Rudy, Wilson Alyce N, Homer Caroline S E, Vogel Joshua P (2021). Clinical guidelines for caring for women with COVID-19 during pregnancy, childbirth and the immediate postpartum period.. Women and birth : journal of the Australian College of Midwives.

